# Recent advances in understanding and managing Paget’s disease

**DOI:** 10.12688/f1000research.19676.1

**Published:** 2019-08-22

**Authors:** Ian R Reid

**Affiliations:** 1 Department of Medicine, Faculty of Medical and Health Sciences, University of Auckland, Auckland, 1142, New Zealand; 2 Auckland District Health Board, Auckland, 1142, New Zealand

**Keywords:** Paget's disease, bisphosphonates

## Abstract

Paget’s disease is a condition which continues to challenge and surprise. The dramatic fall in its incidence over the last three decades has been an enormous surprise, as is the capacity of a single infusion of the potent bisphosphonate, zoledronate, to produce biochemical remission in 90% of patients, remissions which usually persist for many years and raise the possibility of a cure in some patients. However, challenges in its management remain. The trials carried out in Paget’s disease have almost always had biochemical indices as their primary endpoints. From these studies, we also know that bone pain is relieved, quality of life improved, bone histology normalised, and radiological lesions healed. Thus, disease progression is halted. Studies have not been powered to assess whether clinically important endpoints such as fracture and the need for joint replacement surgery are diminished, although these complications are well established as part of the natural history of the condition. Since disease progression is prevented by potent bisphosphonates, it is likely that disease complications will also be prevented. Zoledronate also reduces the frequency of follow-up needed and therefore provides a very cost-effective intervention in those who have symptomatic disease or are at risk of complications.

## Introduction

Paget’s disease of bone manifests as one or more areas of the skeleton with clearly demarcated increased bone turnover. Lesions usually show excess osteoclast activity early in their course, sometimes resulting in lytic abnormalities radiologically. Subsequently, osteoblast over-activity often dominates, producing sclerosis on radiographs and sometimes resulting in local deformity. Bone outside the lesions is normal in Paget’s disease, in contrast to some rare congenital conditions which are sometimes described as “Paget-like”, such as familial expansile osteolysis and idiopathic hyperphosphatasia/juvenile Paget’s disease. These conditions arise from specific genetic mutations not present in Paget’s disease and their clinical presentations and responses to treatment are quite distinct and therefore should not be confused with classic Paget’s disease.

In recent years, there have been two major developments in Paget’s disease. First, its incidence in many countries appears to have dropped dramatically such that it is now a rare condition in almost all regions. Second, it has been demonstrated that a single infusion of intravenous zoledronate produces complete and durable biochemical remission in most patients and so the disease may effectively be cured.

## Aetiopathogenesis

The cause of Paget’s disease remains unclear. There is clearly a genetic component since 10 to 30% of patients have a family history of the condition. Variations in the *SQSTM1* gene—which encodes p62, a protein involved in signal transduction downstream of the receptor activator of nuclear factor kappa B (RANK)—have been demonstrated in about 30% of familial Paget’s patients and in about 10% of sporadic cases. At least seven other genes have been found to be associated with Paget’s disease in genome-wide association studies ^1^ and most are involved in the regulation of osteoclastogenesis. However, pagetic lesions do not demonstrate somatic *SQSTM1* mutations ^2^, the presence of a germline *SQSTM1* mutation does not necessarily result in the clinical development of Paget’s disease ^3^, and the degree of penetrance for these mutations appears to be decreasing over time ^3^. The dramatic fall in incidence and severity of Paget’s disease over the last two to three decades is highly suggestive of an important environmental contribution to the condition. Viral infections and environmental toxins have been suggested but the evidence for these is inconsistent.

The clinical evolution of Paget’s disease provides some insight into its aetiology. Pagetic lesions are clearly circumscribed and usually progress at a rate of about 1 cm/year along or across affected bones ^4, 5^ but are generally considered not to cross to adjacent bones or appear at new sites in the course of the disease. However, Paget’s disease can be transmitted to unaffected bones by bone grafting ^6^, suggesting that it behaves like a benign neoplastic condition. It is often described as primarily a disease of osteoclast excess since this is the striking histological feature and the osteoclasts are highly multi-nucleated. However, increased numbers of osteoblasts are also apparent and indeed are the source of the elevated alkaline phosphatase which is often the means of initially suspecting and diagnosing the condition. Altered gene expression in osteoblasts and bone marrow stromal cells—including increased levels of dickkopf-1, interleukin-1, and interleukin-6—from pagetic bone has been demonstrated, suggesting that the primary abnormality does not necessarily lie in the osteoclasts ^7^. A hypothesis consistent with these findings would be that there is a clone of abnormal cells that regulate osteoclast or osteoblast development in each pagetic lesion and that these cells are unable to spread secondarily via the bloodstream. Possibly, the effect of an environmental factor combined with genetic susceptibility leads to the creation of these abnormal cells. The apparent cure of Paget’s disease following bolus treatment with zoledronate might be attributable to their destruction by the high local concentration of drug achieved as a result of selective uptake of bisphosphonates into pagetic lesions.

## Epidemiology

Paget’s disease is seldom diagnosed before 40 years of age, and prevalence increases with age. It has been found to affect 2 to 3% of the population older than 55 years in the US ^8^ and a slightly higher percentage in some regions of Britain, Spain and Italy but is rare in most of Asia. Several surveys have suggested that prevalence has reduced by about 50% in recent decades in Britain ^9^, Europe ^10^ and New Zealand ^11, 12^. In Lancaster, UK, which used to have one of the highest prevalences, the figure fell by 90% between the 1970s and 2018 ^13^. Patients now present later with less active and less extensive disease ^11, 14, 15^.

## Diagnosis and evaluation

**Figure 1.**
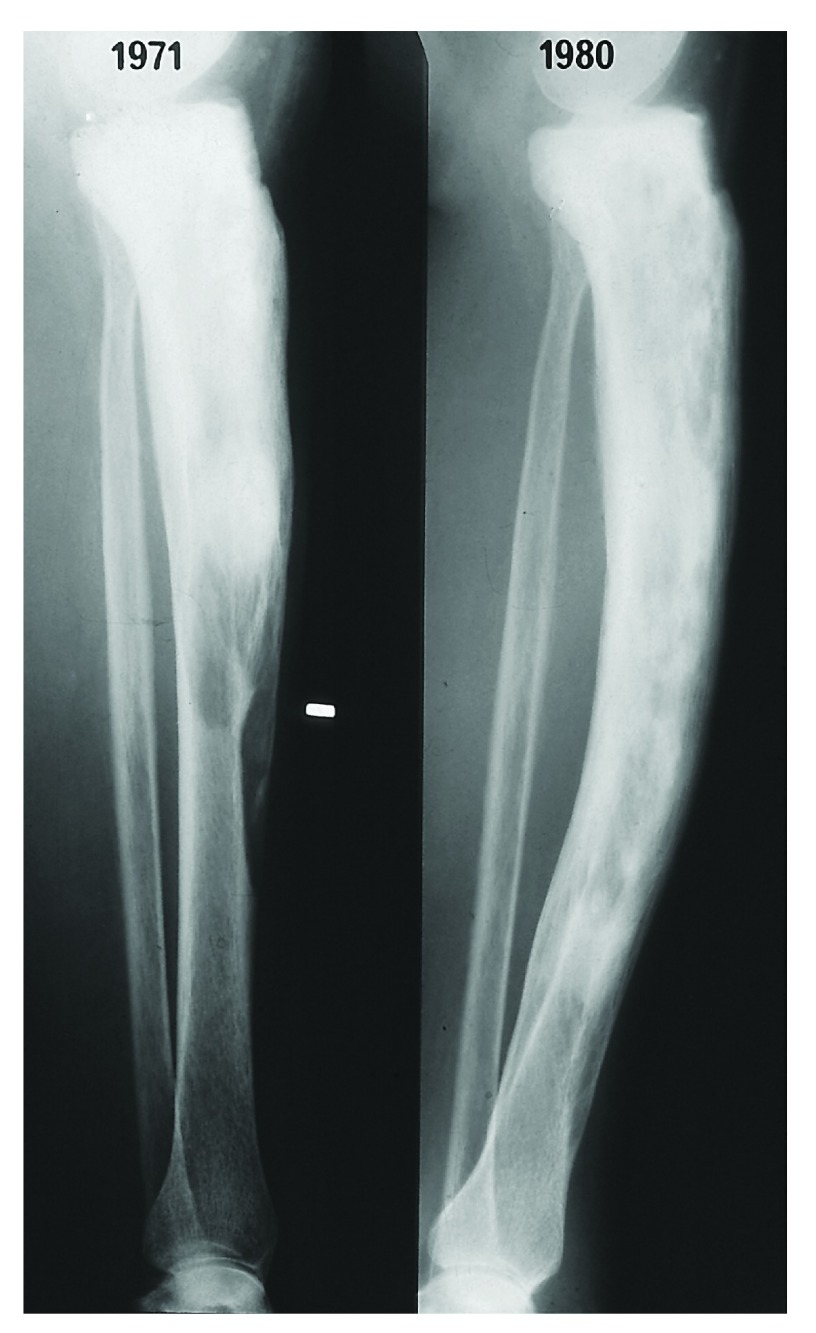
Natural history of Paget’s disease in the tibia. In the absence of therapy, the lytic wedge at the leading edge of the lesion progresses along the bone at about 1 cm/year. Tissue that was previously lytic (left) becomes sclerotic (right) as the predominance of osteoclasts is replaced by osteoblasts. The late H.K. Ibbertson provided this image to the author Ian Reid with permission to use it for publication purposes. © IR Reid.

**Figure 2.**
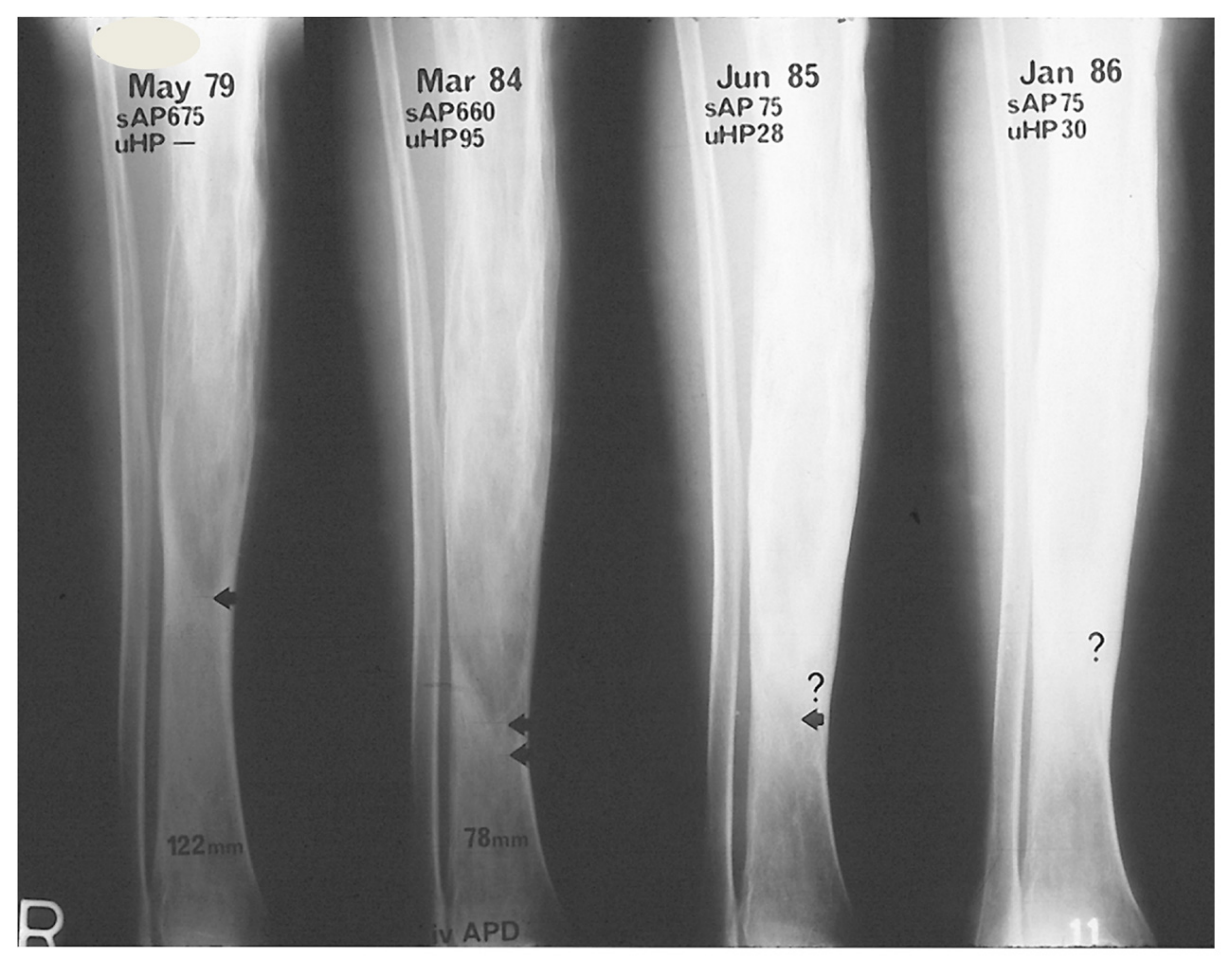
Healing of lytic bone disease in Paget’s disease of the tibia with bisphosphonate. The dates of radiographs are shown, together with the serum alkaline phosphatase (sAP) activities and urine hydroxyproline (uHP) excretions at that time. Between the first two images, no treatment was given, and the lytic wedge (indicated by arrows) progressed. At the time of the second radiograph, a course of pamidronate infusions was administered. One and two years later, the lytic lesion was healed and there was no further progression of pagetic changes along the tibia. iv APD, intravenous 3-amino-1-hydroxypropylidene-1,1-bisphosphonate, or pamidronate. The late H.K. Ibbertson provided this image to the author Ian Reid with permission to use it for publication purposes. © IR Reid.

Diagnosis is usually made on the basis of the typical appearances on plain radiographs. Abnormalities include evidence of osteolysis, coarsening of trabecular pattern, sclerosis, and bone deformity ( Figure 1 and Figure 2). Pagetic lesions are usually clearly demarcated from adjacent normal bone and they progress within an affected bone but do not migrate to adjacent bones. Active pagetic lesions are usually clearly visible on radionuclide scans, but appearances are less specific than those of plain radiographs and therefore should not be the sole basis for diagnosis. However, scintigraphy provides an excellent determination of disease distribution and local activity and therefore complements plain radiographs. Computed tomography or magnetic resonance imaging can also be helpful if the diagnosis is uncertain but neither is usually required.

It is usual to quantify disease activity through measurement of bone turnover markers. Total alkaline phosphatase is the most widely used assay for this purpose and is satisfactory as long as there is no disturbance of liver function. Serum procollagen type 1 N-terminal propeptide (PINP) and bone-specific alkaline phosphatase are marginally more sensitive and specific but are less widely available and more expensive ^16, 17^. Bone resorption markers are also markedly elevated in active Paget’s disease, but the most commonly used marker in other conditions, β-CTX, performs inconsistently in Paget’s disease and can show paradoxical responses to therapy. For these reasons, the total alkaline phosphatase or PINP is usually preferred.

It is important to note that when there is only a single site of skeletal involvement, biochemical markers can be normal despite the presence of radiological progression and active scintigraphic abnormality. Therefore, it is inappropriate to conclude that normal biochemistry indicates the absence of active Paget’s disease when skeletal involvement is limited.

## Treatment

There is widespread agreement that a single intravenous injection (5 mg) of zoledronate (also known as zoledronic acid) is the first-line therapy for Paget’s disease ^18, 19^. This is based on its superiority over other agents in inducing biochemical remission and its unprecedented duration of maintenance of those remissions. Thus, in the phase 3 trial programme of zoledronate, which involved 349 patients with very active Paget’s disease (mean baseline alkaline phosphatase of 427 U/L), alkaline phosphatase was normalised with zoledronate in 89%, in comparison with only 58% in a comparator group randomly assigned to risedronate. Quality-of-life scores improved in the zoledronate groups but not with risedronate ^20^. During follow-up over 6 years, alkaline phosphatase remained normal in 79% of zoledronate-treated patients who initially achieved remission but in only 55% of risedronate-treated patients who initially normalised alkaline phosphatase ^21^. The majority of zoledronate-treated patients showed stable bone markers throughout follow-up and no trend towards relapse. Quality of life remained superior in the zoledronate group during follow-up.

We have been using zoledronate as first-line therapy in Paget’s disease for more than 15 years and our clinical experience has mirrored the trial findings; a recent survey of 107 treated patients showed that all normalised serum PINP after zoledronate and only 14% had increases in PINP to values of more than 80 μg/L over 10 years of follow-up ^22^. In this cohort, mortality from other causes over the 10 years was 55%, so most patients would never require re-treatment. These high response rates and the stability of the resulting remissions greatly reduce the frequency of follow-up needed. My follow-up practice is to measure serum alkaline phosphatase or PINP at 6 months since those who achieve values of less than 80 U/L or less than 40 μL, respectively, have 6-year relapse rates of less than 10% ^21^. I re-assess patients at 3 to 5 years. If markers remain normal, I carry out a bone scintigram at year 5 since this is a much more sensitive measure of disease activity than markers. Our experience indicates that among those with normal markers 5 years after zoledronate, a third have normal scans, a third have evidence of only trivial disease activity not requiring intervention, and the balance may merit re-treatment ^23^. Whether those with normal scans at 5 years are cured is not known, but the likelihood of these elderly patients requiring future treatment is very low.

Criteria for re-treatment are not clearly established, regardless of whether markers are elevated. The Paget’s Randomized trial of Intensive versus Symptomatic Management (PRISM) suggested that pressing hard to normalise markers that were only marginally elevated did not make a difference to clinical outcomes, other than analgaesic use, although this study was not powered to assess most of the endpoints reported ^24, 25^. However, more aggressive therapy is required in some circumstances, such as in the management of paraplegia from spinal Paget’s disease and in achieving healing of lytic lesions in the femur and tibia, where it is important to confirm radiological healing, not just normalisation of turnover markers.

Oral alendronate (40 mg/day for 6 months) ^26, 27^ and risedronate (30 mg/day for 2 months) ^28^ have also been shown to produce substantial suppression of biochemical indices in Paget’s disease; risedronate shows reduction in pain, and alendronate restores normal histology and heals radiological lesions. However, neither achieves such high remission rates as zoledronate, and the offset of effect for risedronate is considerably more rapid ^21^. For the latter reason, alendronate is to be preferred if an oral agent is used. The duration of treatment chosen in the key clinical trials was arbitrary and so individual patients may require longer or shorter initial courses to achieve remission. Many other bisphosphonates have demonstrated a range of efficacies but are not widely available and therefore are little used.

Denosumab has also been used for treatment of Paget’s disease in a few cases. Biochemistry but not scintigraphy has been normalised, and 6-monthly dosing appears to be necessary ^29^. It may be preferable to the only other non-bisphosphonate drug available, calcitonin injections, which seldom normalise biochemistry, require daily injections, and commonly cause flushing and nausea.

## Recent guidelines

In recent years, guidelines have been published by the US Endocrine Society ^18^ and by the UK Paget’s Association ^19^, among others. They agree that the incidence of Paget’s disease has declined substantially, their approach to patient evaluation is similar, and both regard intravenous zoledronate as the first-line therapy. They differ with respect to recommendations in areas where there is not clear guidance from clinical trials, such as for the prevention of pagetic complications. As pointed out in a recent editorial ^30^, the Endocrine Society guideline provides advice based on expert opinion for those common clinical questions not addressed in clinical trials, whereas the UK guideline implies that no intervention other than for those patients with pagetic bone pain is needed.

In many ways, this is a philosophical difference as to how we should act in the absence of definitive trials with clinical endpoints and what other evidence can guide decision-making. There is a wealth of clinical data from the pre-bisphosphonate era establishing the natural history of Paget’s disease, demonstrating that this is a progressive condition. Lytic lesions progress at a rate of 1 cm/year ^4, 5^ with accompanying deformity ^31^ ( Figure 1 and Figure 2). In contrast, treatment with potent bisphosphonates restores normal lamellar bone in place of the woven bone characteristic of Paget’s ^26^, results in healing of radiographic lesions ^26, 32– 34^, reduces pain ^19, 20, 28^ and improves quality of life ^20, 21^. Thus, disease progression can be halted on the basis of histological and radiological evidence. Since it is the progression of lesions that results in the development of deformity and since this progression can be halted with potent bisphosphonates, it seems highly probable that deformity and its sequelae (pain, osteoarthritis, nerve entrapment, fractures and paraplegia) are also prevented by these interventions. Pagetic fractures also occur through lytic lesions and so the demonstration of healing of these lesions with bisphosphonates ^26^ suggests that this fracture type will also be prevented. Some pagetic complications, such as paraplegia, are too rare to ever be the subject of trials, yet a body of case reports indicates that surgical management often has disastrous outcomes whereas bisphosphonates can be curative ^35^. This hard-won clinical evidence must inform our patient management and not just be discarded because it is not a clinical trial. The diminishing incidence and severity of Paget’s disease, together with the availability of an intervention that provides complete and life-long disease control after a single treatment, make it unlikely that the trials that would be necessary to address the questions of prevention of fractures, osteoarthritis and so on will ever be undertaken and so clinical management needs to be informed by clinical experience and an understanding of the natural history of the disease as well as the valuable insights we have gained in the last 40 years of clinical trials.
